# Assessment of Patients with a DePuy ASR Metal-on-Metal Hip Replacement: Results of Applying the Guidelines of the Spanish Society of Hip Surgery in a Tertiary Referral Hospital

**DOI:** 10.1155/2014/982523

**Published:** 2014-11-09

**Authors:** Jenaro Fernández-Valencia, Xavier Gallart, Guillem Bori, Sebastián Garcia Ramiro, Andrés Combalía, Josep Riba

**Affiliations:** Hip Unit, Department of Orthopaedic Surgery and Traumatology, Hospital Clínic, Universitat de Barcelona, C/Villarroel 170, 08036 Barcelona, Spain

## Abstract

The prognosis associated with the DePuy ASR hip cup is poor and varies according to the series. This implant was withdrawn from use in 2010 and all patients needed to be assessed. We present the results of the assessment of our patients treated with this device, according to the Spanish Society of Hip Surgery (SECCA) algorithm published in 2011. This retrospective study evaluates 83 consecutive ASR cups, followed up at a mean of 2.9 years. Serum levels of chromium and cobalt, as well as the acetabular abduction angle, were determined in order to assess their possible correlation with failure, defined as the need for revision surgery. The mean Harris Hip Score was 83.2 (range 42–97). Eight arthroplasties (13.3%) required revision due to persistent pain and/or elevated serum levels of chromium/cobalt. All the cups had a correct abduction angle, and there was no correlation between elevated serum levels of metal ions and implant failure. Since two previous ASR implants were exchanged previously to the recall, the revision rate for ASR cups in our centre is 18.2% at 2.9 years.

## 1. Introduction

In September 2010, the Spanish Agency of Medicines and Medical Devices issued an alert calling for the withdrawal from use and recall of the ASR Hip Resurfacing System and ASR XL Acetabular System total hip replacement, both manufactured by DePuy International Ltd. The basis for the recall was a higher-than-normal failure rate and the possibility of metal debris (cobalt-chromium alloy) being deposited in the surrounding tissues and with possible toxic implications related to cobalt release. The Spanish Society of Hip Surgery (SECCA) published an algorithm for the assessment of patients who had been treated with one of these devices [1]. This paper reports the results obtained in a series of patients who were evaluated with this algorithm as a response to the recall.

## 2. Material and Method

In December 2010, a letter was sent out to all patients who had undergone a hip arthroplasty at the Hospital Clinic in Barcelona and who had been fitted with a DePuy ASR cup. This involved a total of 92 cups in 83 patients (10 women and 73 men), with a mean age of 51 years (range 24–66, SD 10). Nine of these patients had received a bilateral implant. Their mean body mass index was 28 kg/m^2^ (range 21.1–36.2).

Five patients did not respond to the letter, and three had died for reasons unrelated to the hip surgery. This left a total of 83 DePuy ASR cups, 60 of which corresponded to resurfacing and 23 to the use of the short-stem Proxima implant. Mean time at follow-up was 2.9 years (range 0.9–5.5, SD 1.05). In the original indications for surgery, patients with chronic renal insufficiency, women of a fertile age, and patients with a known allergy to metals were all excluded.

All the surgical interventions used the modified Hardinge (lateral) approach [2], with the patient in the lateral decubitus position. A complete anterior capsulectomy was performed prior to dislocation of the femoral head. The acetabulum and femur were prepared in accordance with the standard technique. As part of our routine protocol for hip arthroplasty, all the patients received 1.5 g cefuroxime at induction of anaesthesia, with a further dose two hours later if required by the duration of surgery. Low molecular weight heparin was used for antithrombotic prophylaxis. For all the revised hips, at least one tissue sample was submitted for histological study. Two cases were metal-stained at some degree. All of the tissues were fixed in 10% formalin immediately after removal. Samples were examined by two different pathologists and were assessed for lymphocytes, macrophages, plasma cells, giant cells, necrosis, and metal wear particles.

During the follow-up assessment visit, all patients provided informed consent for their case to be studied. In all cases, the Harris Hip Score [3] was obtained, and radiology and laboratory tests were performed, including determination of serum chromium (Cr) and cobalt (Co) levels. The latter were measured by means of mass spectrometry (Perkin Elmer AA600 graphite furnace atomic absorption spectrometer), with the following being considered as elevated levels: >5 *μ*g/L for Co and >2 *μ*g/L for Cr. The Harris Hip Score was categorized using the criteria described by Marchetti et al. [4]: <70, poor; 70–79, fair; 80–89, good; and 90–100, excellent.

The standardized anterior-posterior radiographs of the pelvis were taken (with the foot internally rotated) before and after surgery. All radiographs were assessed to check that the hip presented a similar degree of rotation: this was done by noting the appearance of the lesser trochanter in the preoperative radiograph and searching for the best correspondence among the available postoperative radiographs. The acetabular abduction angle, as well as the presence of osteolysis or signs of loosening, was determined. The acetabular angle was measured as the angle between the horizontal tear drop line and the axis of the acetabular component [5].

In 13 patients, computed tomography (CT) was performed as a complementary evaluation to the above exams. This was done in the Department of Radiology using a Somatom Plus IV scanner (Siemens, Erlangen, Germany), with CT parameters being optimized using Siemens' own software. Images were obtained of the whole prosthesis, from the upper part of the acetabulum to the lower part of the femoral implant, using helical acquisition with the following parameters: kV = 120, mAs = 200, slice thickness = 3 mm, pitch = 1.5, and reconstruction space = 3 mm. A bone reconstruction algorithm and wide window were used to accentuate the border of bone structures, the aim being to minimize artefacts due to the metal prosthesis. The images were also reconstructed using a soft tissue algorithm.

Data were analysed using the statistical software IBM SPSS Statistics version 20. In addition to evaluating whether Cr and Co levels increased over time, a Spearman test was used to assess the possible relationship between the acetabular abduction angle and levels of these two metal ions. The group of patients who underwent revision surgery was also compared with the group who did not in terms of sex, age, Cr and Co levels, acetabular abduction angle, cup size, and time since primary surgery. The chi-squared test was used to assess differences by sex, while the Mann-Whitney *U* test was applied for the remaining variables. The level of statistical significance was set at *P* ≤ 0.05.

## 3. Results

The mean Harris Hip Score (83.2, range 42–97) was regarded as good. The mean acetabular abduction angle was 39.8° (range 15–52, SD 5.5), and there were no cases of loosening or osteolysis.

Serum cobalt levels were elevated (≥5 *μ*g/L) in 10 patients (1 bilateral), with a mean in this subgroup of 12.3 *μ*g/L (range 5–56, SD 14.2). Serum chromium levels were high (≥2 *μ*g/L) in 14 patients (4 bilateral), with a mean in this subgroup of 7.3 *μ*g/L (range 2.1–40, SD 9.4).

The CT images obtained from 13 patients revealed no relevant findings, except for 2 patients with trochanteric bursitis, 1 with excess fluid in the iliopsoas bursa, 1 with a fluid collection anterior to the femoral neck, and 1 with fluid/swelling around the greater trochanter.

Cr and Co levels did not vary significantly over time (*P* = 0.396), and no relationship was observed between these levels and the acetabular abduction angle (*P* > 0.05). There were no differences between patients who underwent revision surgery and those who did not in terms of sex (*P* = 0.653), age (*P* = 0.790), levels of Cr (*P* = 0.727) and Co (*P* = 0.447), acetabular abduction angle (*P* = 0.259), cup size (*P* = 0.087), or time since primary surgery (*P* = 0.819).

**Figure 1:**
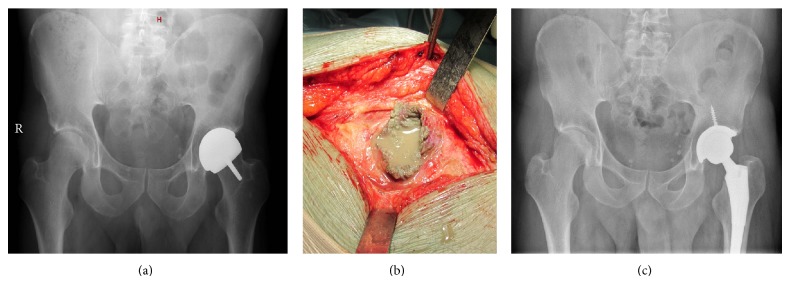
(a) Anterior-posterior radiograph of the pelvis prior to revision surgery, showing no relevant radiological findings related to the implant; (b) intraoperative appearance of the pseudotumour during revision surgery; and (c) radiograph of the same patient 6 months after revision.

Eight resurfacing implants required revision due to the presence of pain and/or elevated serum levels of chromium/cobalt. The intraoperative findings were of no relevance in six cases; in one case a solid pseudotumour was identified, while another patient presented with metallosis and a fluid-filled pseudotumour (Figure 1). The histology of the case of the pseudotumour showed lymphoplasmacellular infiltration and presence of macrophages and multinucleated giant cells depicting a foreign body reaction. There was also presence of polymorphonuclear cells at low ratio (less that 5 per wide microscopic field (×40)). The histology of the metallosis and fluid-filled pseudotumour showed severe pigmentary histiocytic reaction. Two other samples without the clinical appearance of fluid-filled pseudotumour showed also severe pigmentary histiocytic reaction. For the rest of the histologies, moderate to low rate of inflammatory cells was observed without signs of metallosis.

Added to these eight revisions after the recall, two previous ASR implants were exchanged previously to the recall: as a result, the revision rate for ASR cups in our centre is 18.2% at 2.9 years.

## 4. Discussion

Since the original findings of the Australian National Joint Replacement Registry [6], more recent series [7, 8] have confirmed the elevated failure rates associated with the DePuy metal-on-metal ASR system, both in the case of the resurfacing and in the case of the large-diameter prosthesis. This failure rate, followed by the subsequent recall of the implant, has received considerable attention in the literature [9]. One of the main concerns relates to the possible effects of the deposition of Cr and Co debris, both locally and in the bloodstream, there being three reported cases of cobalt toxicity associated with this prosthesis [10, 11].

The revision rate in our centre was found to be 18.2% at a mean follow-up of 2.9 years. In their review of the Australian National Registry, De Steiger et al. found that the cumulative revision rate at five years for arthroplasties involving the ASR Hip Resurfacing System was 10.9% (95% CI, 8.7% to 13.6%), compared with 4% (95% CI, 3.7% to 4.5%) for all other resurfacing prostheses [6]. Although this rate is high in itself, more recent series have reported results similar to our own, that is, even higher rates at shorter follow-up. For example, Bernthal et al. reported a revision rate at three years of 17.1% [8]. In their series, 12 implants that functioned correctly during the first postoperative year subsequently failed within 3 years, due to pain (7 cases), loosening (3 cases), and squeaking (2 cases). The remainder presented persistent pain without radiological evidence of loosening. The radiographs of patients in our series revealed no relevant findings, and the main cause of revision was the presence of persistent pain.

There is currently no consensus regarding what constitutes acceptable or clearly toxic levels of metal ions in the bloodstream of patients fitted with orthopaedic implants, although it has been suggested that below 2 *μ*g/L is acceptable in the case of Co [12]. In fact, all implants may release these ions regardless of the friction pair. For example, a study of 41 knee arthroplasties found levels of Cr and Co to be 0.92 and 3.28 *μ*g/L, respectively, in unilateral prostheses, and 0.98 and 4.28, respectively, in bilateral implants [13]. A case of cobalt intoxication has also been reported in a patient who did not originally have a metal-on-metal implant [14].

Reported levels of Co and Cr ions vary depending on the series. One multicentre study involving a 24-month follow-up of 77 patients implanted with a unilateral resurfacing prosthesis found that ion levels were generally low and had stabilized by 3 months [15]. Six patients had abnormally high metal ion levels, and all of them also had a cup abduction angle above 55°. In another study, Desy et al. reported that a smaller ASR cup diameter was associated with higher levels of Cr and Co, as well as with larger acetabular inclination [16]. Randelli et al. also found that a cup inclination angle greater than 50° led to increased concentrations of metal ions, although this angle was only present in three of their patients [17]. The study by de Haan et al. similarly observed a greater release of metal ions with abduction angles above 55° [18]. Langton et al. compared the metal ion concentrations associated with two kinds of resurfacing device, the ASR (DePuy) and the BHR (Smith & Nephew), and again found that concentrations were higher with larger inclination angles, although this was only the case for implants with a smaller cup diameter [19]. In our series, the acetabular abduction angle never exceeded 55°, and yet there were 10 cases of elevated Co levels and 14 cases of high Cr levels. This illustrates how it is not only the cup position which is important, but also the design and characteristics of the implant.

Our patients who required prosthesis revision showed no radiological signs of osteolysis or loosening, and their implants were correctly positioned. In one case a solid pseudotumour was observed, while another patient presented with metallosis and a fluid-filled pseudotumour. The failure of ASR prostheses has been attributed to their design, specifically to the increased risk of edge loading [18, 19], a problem that particularly affects smaller-diameter components [20]. Implant failure in the absence of radiological changes may be explained by soft tissue lesions secondary to the release of metal ions, a phenomenon that has been termed “adverse reaction to metal debris” (ARMD) and which includes various changes such as pseudotumours [21], aseptic lymphocyte-dominated vasculitis-associated lesions (ALVAL) [22], and metallosis [23]. AMRD is not considered to correlate to the amount of wear [24] and may be linked to a type IV hypersensitivity reaction [12].

## 5. Conclusions

Although we agree that it is important that acetabular abduction does not exceed 55°, this does not appear to have been a key factor in the failure of our ASR prostheses, since none of the patients in this series had an implant over that angle. The survival rate of the ASR cup at a mean of 2.9 years was 100% among patients with a short-stem Proxima implant and 82.8% in those with a resurfacing prosthesis.
